# Validation of a patient satisfaction survey of the Teleneurology program in Chile

**DOI:** 10.1186/s13104-019-4358-1

**Published:** 2019-06-25

**Authors:** Freddy Constanzo, Paula Aracena-Sherck, Juan Pablo Hidalgo, Maritza Muñoz, Gerardo Vergara, Cristóbal Alvarado

**Affiliations:** 1 Neurology Unit, Hospital Las Higueras, Alto Horno 777, Talcahuano, Chile; 2 Medical Program in Adult Neurology, School of Medicine, Universidad Católica de la Santísima Concepción, Concepción, Chile; 3 Department of Science, School of Medicine and Science, Universidad San Sebastián, Concepción, Chile; 4 Department of Statistics, School of Sciences, Universidad del Bío-Bío, Concepción, Chile; 5 Department of Public Health, School of Medicine, Universidad Católica de la Santísima Concepción, Concepción, Chile; 6 Unit of Teleprocesses, Hospital Las Higueras, Talcahuano, Chile; 7 Department of Basic Sciences, School of Medicine, Universidad Católica de la Santísima Concepción, Concepción, Chile

**Keywords:** Validation studies, Satisfaction survey, Telemedicine, Teleneurology, Adult neurology

## Abstract

**Objective:**

Telemedicine arises as an attractive intervention for reducing the waiting time for appointments with medical specialists in Chile. Successful implementation of this technology requires safeguarding the patient/clinician trust relationship; however, no studies have been conducted to evaluate quality perception of a telemedicine program in Chile. To assess patient satisfaction with the Teleneurology program at the Hospital Higueras Talcahuano (HHT), addressing patient/clinician trust relationship.

**Results:**

A perception survey was constructed with 23 questions, distributed into 5 key areas (items) of user satisfaction. Its face validity was performed by five neurology specialists from the Teleneurology unit of HHT. The survey was applied to 167 patients of the HHT, recruited between 2018 and 2019, for conducting a pilot cross-sectional descriptive study to assess internal consistency (Cronbach alpha) and reliability (factorial analysis of main components). The survey showed an internal consistency of 0.88. Removing any of the items maintained its reliability in values over 0.8. All items showed point biserial correlations greater than 0.30. Overall, the survey constructed and evaluated in this study showed high internal consistency and reliability values, which will allow its application in further studies of quality assessment of the Teleneurology unit of HHT.

**Electronic supplementary material:**

The online version of this article (10.1186/s13104-019-4358-1) contains supplementary material, which is available to authorized users.

## Introduction

The lack of timely access to health care is a growing issue worldwide, particularly in the population with a disability, such as neurological patients [1] The deficit in the care of neurological patients can be explained by: (i) lack of resources to improve the primary care system [2], (ii) deficiencies in access to health care due to disability of patients or the distances they must travel to obtain adequate health care [3], and (iii) a deficit in the number and distribution of neurologist specialists. Telemedicine, which involves the remote access of a patient with a medical practitioner via the internet, has the potential to address some of these deficits. Thus, Teleneurology interventions are being implemented and evaluated in several regions of the globe [4].

The Teleneurology program implemented in 2015 at the Hospital Las Higueras of Talcahuano (HHT), belonging to the Healthcare Unit of Talcahuano (SST, Spanish acronym for *Servicio de Salud Talcahuano*) of the Ministry of Health, has pioneered addressing the access to adult neurology care with specialists in the country, through a technology that combines the simultaneous live care by a general practitioner in situ, and the remote primary care office by a neurology specialist in specifically designed HHT facilities. All patients have an active participation, making them permanently involved in the delivery of information, complete evaluation and resolution of the health problem. For each video session in the Teleprocess unit (synchronous), a referral and reference web platform are used, which is integrated with the hospital electronic clinical record with an HL7 standard and has an SSL (Secure Sockets Layer) security server for encryption.

Although telemedicine can address the issue of timely access to health care, it is possible that this type of interventions can adversely impact on the quality of the specialist’s relationship with his patient. However, very few tools evaluating the perception of patients for their health care in Teleneurology programs exist, and all of them are in English [4], leaving the entire Spanish speaking population without an opportunity to evaluate quality of care. Given that the HHT Teleneurology program has been in operation for 4 years, it became necessary to assess its patient satisfaction, in order to investigate possible deficiencies that may exist in the generation of the patient-specialist physician trust bond [5–8]. To this end, a perception survey was elaborated for the first time in Chile to evaluate the patient satisfaction of a Teleneurology unit. A questionnaire of 23 items was constructed to assess 5 dimensions of patient perception: general perception of satisfaction, perception of telemedicine, perception of the patient’s active participation in their own well-being, perception of the convenience of the system and perception of interaction with the doctor specialist. The present study shows the results of the evaluation of this survey, applied in the form of a pilot cross-sectional study in a cohort of 167 patients of the Teleneurology program of the HHT, recruited from September 2018 to February 2019. The survey was evaluated in terms of content, reliability, discrimination and difficulty, for future evaluation of this and other Teleneurology programs in the region, in terms of patient perception of quality.

## Main text

### Methods

#### Design and population

Cross-sectional study in a cohort of 167 patients of the HHT Teleneurology program. Participants were selected among the patients who were treated within this program between September 2018 and February 2019, through a non-probabilistic sampling (by volunteering). Participants had to meet the inclusion and not meet the exclusion criteria described in the reference protocol of the Teleneurology program (exempt resolution number 4889 of December 31, 2015), issued by the SST of the Ministry of Health (Additional file 1: Table S1). It should be noted that persons with mental disabilities or who were minors were also excluded, as specified in the Chilean Law 28584, article 28. The research protocol was approved by the Scientific Ethics Committee of the SST of the Ministry of Health. All participants signed a written informed consent.

#### Survey design

A patient satisfaction questionnaire was designed and constructed in Spanish, consisting of a total of 23 questions with closed responses on a single Likert scale (totally disagree, disagree, neither agree nor disagree, agree, totally agree). This survey was aimed to evaluate the general perception of patient satisfaction (questions 1 and 2), perception of telemedicine care (questions 3 to 5), perception of the patient’s active participation in their own well-being (questions 6 to 13), perception of convenience of the system (questions 14 to 18) and perception of interaction with the medical specialist (questions 19 to 23). Most questions (20 out of 23) were designed based on published English language satisfaction surveys in telemedicine [9–14]. On the other hand, three questions were specifically designed for the Teleneurology program of HHT, given that the medical care of this program involves the participation of a general practitioner who accompanies the patient during the remote medical appointment with the neurology specialist (questions 13, 22 and 23) (Additional file 1: Table S2). The survey questionnaire, with a maximum score of 115 points, was graded in terms of satisfaction: very low (under or equal to 23 points), low (24 to 46 points), moderate (47 to 69 points), high (70 to 92 points), and very high (93 to 115 points). The questionnaire was self-administered in order to safeguard the anonymity of the study participant.

#### Survey evaluation and statistical analysis

Face validity of the survey, in terms of language and particulars of the neurology practice was assessed by five neurology specialists from the Neurology Unit at the HHT. A descriptive analysis of normality of the sample (Kolmogorov–Smirnov) was conducted. The internal consistency was evaluated by Cronbach’s alpha test, which suggests the following scale for alpha coefficients: excellent (> 0.9), good (> 0.8), acceptable (> 0.7), questionable (> 0.6), poor (> 0.5), and unacceptable (< 0.5) [11]. Difficulty and discrimination of the instrument were evaluated by index of difficulty and specific biserial correlation, respectively. Finally, suitability of the data for structure detection was conducted using Kaise–Meyer Olkin measure of sample adequacy and Bartlett’s sphericity test. All analyses were carried out in SPSS, version 25.0. Statistical significance was established at p < 0.05.

### Results

#### Patient cohort description

Of the total number of respondents, 71.86% were women, while the rest were men. Over 97% of men and women showed a very high level of satisfaction with respect to the care received through the Teleneurology program of the HHT. According to Kolmogorov–Smirnov’s analysis, the sample did not exhibit a normal distribution (*p* < 0.05). Statistically significant differences were also found in the distribution of the population according to sex (*p* < 0.05).

#### Survey evaluation

Face validity of the survey was conducted by five local neurology specialists at the HHT. This allowed for the wording of each question to conform both the Spanish language and the pertaining to the neurology practice of medicine (not shown). The questionnaire applied to the voluntary patients was already in its revised form (see Additional file 1: Table S2 for the original Spanish questions).

The reliability analysis of the questionnaire (Table 1) showed that internal consistency was high, with a Cronbach’s alpha of 0.88. Only the consistency of the item on the “general perception of satisfaction” was unacceptable (Cronbach’s alpha 0.47), while those of the items “perception about the active participation of the patient in their own well-being”, “perception about the interaction with the specialist”, “perception of Telemedicine”, and “convenience of the system” were acceptable (Cronbach’s alphas 0.79, 0.75, 0.73, and 0.79, respectively).

**Table 1:** Analysis of internal consistency per question of the user satisfaction survey (n = 167)

Question number	Variables^a^	α when the question is deleted
	Total survey with 23 questions	0.88
1	I am satisfied with the care received in Telemedicine	0.87
2	My family is satisfied with the care received in Telemedicine	0.88
3	Telemedicine helps me to know my state of health	0.88
4	Telemedicine helps me know how to improve my health status	0.88
5	Telemedicine allows me to better follow the recommendations and indications of my specialist doctor	0.87
6	I felt comfortable talking to my specialist doctor through a camera and a microphone	0.87
7	Talking to my specialist doctor. through a camera and a microphone. was as effective as in person	0.87
8	During my Telemedicine care it was easy for me to explain my health problem to my specialist doctor	0.87
9	My specialist doctor has identified my health problem through Telemedicine	0.87
10	I have been informed of my right to privacy of my personal and medical information included in Telemedicine	0.88
11	I trust that my personal information and privacy will be protected after my attention by Telemedicine	0.88
12	The quality of the image and sound were adequate to talk to my specialist doctor	0.88
13	The general doctor who accompanied me in person helped me during my Telemedicine consultation	0.88
14	My attention by Telemedicine was helpful to me	0.87
15	The time with a specialist is faster by Telemedicine	0.88
16	I prefer Telemedicine because it is easier to go to the doctor’s office than to go to the hospital	0.87
17	I prefer Telemedicine because it is cheaper to go to the office than to go to the hospital	0.88
18	For my future controls I will prefer to continue using Telemedicine	0.88
19	My specialist doctor was able to answer my questions through Telemedicine	0.87
20	My specialist doctor showed concern in solving my health problem during Telemedicine care	0.88
21	I trust the instructions of my specialist doctor during my Telemedicine care	0.88
22	The general practitioner who accompanied me in person during the Telemedicine service was able to answer my questions	0.88
23	The general practitioner who accompanied me in person during the Telemedicine care could answer the questions of my specialist doctor	0.88

^a^Original Spanish questions in Additional file 1: Table S2

The mean of patient satisfaction score in the total of the surveyed population in this pilot study (Table 2) was 110.4 ± 5.98 points; for men it was 111.1 ± 4.84 and for women of 110.1 ± 6.37. The elimination of any of the questionnaire items led to maintaining its reliability as high (Cronbach’s alpha > 0.8), regardless of the element that is removed (Additional file 1: Table S3).

**Table 2:** Descriptive data (mean and SD) for total population and by gender; and reliability analysis (α-Cronbach) of the patient the satisfaction survey

Question number	Variables*	Total (167)		Women (120)		Men (47)		α
		Mean	SD	Mean	SD	Mean	SD	
	*Total survey*	110.40	5.98	110.10	6.37	111.13	4.84	*0.88*
	*General perception of satisfaction*							0.47
1	In general. I am satisfied with the care received in Telemedicine	4.86	0.38	4.84	0.41	4.89	0.31	
2	In general. my family is satisfied with the care received in Telemedicine	4.51	0.76	4.47	0.78	4.61	0.71	
	*Perception of Telemedicine*							0.79
3	Telemedicine helps me to know my state of health	4.78	0.50	4.78	0.48	4.80	0.54	
4	Telemedicine helps me know how to improve my health status	4.71	0.54	4.75	0.47	4.61	0.68	
5	Telemedicine allows me to better follow the recommendations and indications of my specialist doctor	4.81	0.45	4.79	0.47	4.85	0.42	
	*Perception of the patient’s active participation in their own well-being*							0.75
6	I felt comfortable talking to my specialist doctor through a camera and a microphone	4.76	0.51	4.71	0.56	4.89	0.31	
7	Talking to my specialist doctor. through a camera and a microphone was as effective as in person	4.70	0.62	4.64	0.67	4.87	0.40	
8	During my Telemedicine care it was easy for me to explain my health problem to my specialist doctor	4.74	0.59	4.71	0.63	4.83	0.49	
9	My specialist doctor has identified my health problem through Telemedicine	4.75	0.59	4.69	0.65	4.89	0.38	
10	I have been informed of my right to privacy of my personal and medical information included in Telemedicine	4.80	0.54	4.84	0.43	4.70	0.76	
11	I trust that my personal information and privacy will be protected after my attention by Telemedicine	4.85	0.39	4.85	0.38	4.87	0.40	
12	The quality of the image and sound were adequate to talk to my specialist doctor	4.80	0.51	4.78	0.49	4.85	0.56	
13	The general doctor who accompanied me in person helped me during my Telemedicine consultation	4.91	0.31	4.89	0.34	4.98	0.15	
	*Perception of the convenience of the system*							0.73
14	In general. my attention by Telemedicine was helpful to me	4.89	0.40	4.86	0.45	4.98	0.15	
15	The time with a specialist is faster by Telemedicine	4.83	0.48	4.82	0.48	4.85	0.47	
16	I prefer Telemedicine because it is easier to go to the doctor’s office than to go to the hospital	4.83	0.46	4.85	0.40	4.78	0.59	
17	I prefer Telemedicine because it is cheaper to go to the office than to go to the hospital	4.68	0.63	4.65	0.66	4.76	0.57	
18	For my future controls I will prefer to continue using Telemedicine	4.69	0.67	4.67	0.68	4.76	0.64	
	*Perception of interaction with the specialist doctor*							0.79
19	My specialist doctor was able to answer my questions through Telemedicine	4.89	0.33	4.88	0.32	4.91	0.35	
20	My specialist doctor showed concern in solving my health problem during Telemedicine care	4.91	0.31	4.90	0.30	4.96	0.29	
21	I trust the instructions of my specialist doctor during my Telemedicine care	4.92	0.30	4.92	0.28	4.93	0.33	
22	The general practitioner who accompanied me in person during the Telemedicine service was able to answer my questions	4.89	0.36	4.89	0.36	4.91	0.35	
23	The general practitioner who accompanied me in person during the Telemedicine care could answer the questions of my specialist doctor	4.93	0.28	4.93	0.28	4.93	0.25	

The five key areas (items) of user satisfaction are shown in italicface letters

* Original Spanish questions in Additional file 1: Table S2

Difficulty of the instrument’s questions was assessed by using the difficulty index, it should be noted that none of the items showed an index lower than 0.10; therefore, no items was considered for discarding (Additional file 1: Table S4).

The patient satisfaction survey analysed in this pilot study showed very high levels of patient satisfaction (> 97%, n = 167). The use of Cronbach’s alpha coefficient allowed for evaluation of the reliability of the elaborated instrument. Results showed that the patient satisfaction survey of the HHT Teleneurology program shows a good general reliability (Alpha = 0.88, Additional file 1: Table S5), with a good coherence between its questions.

The analysis of discrimination and difficulty showed that the instrument presents a high index of discrimination, except for questions 22 and 23, which could be revised and rewritten for future analysis, as shown in Additional file 1: Table S5.

Finally, a factorial analysis of the originally designed survey suggested a new questionnaire order in 5 item areas as well as the elimination of one outlier question (Table 3). The new suggested final questionnaire has 22 questions broken down into the following item areas: (i) quality of care; (ii) patient satisfaction; (iii) patient’s relationship with the medical team; (iv) patient’s relationship with the specialist doctor and (v) trust in the Teleneurology unit, whose reliability indexes were 0.80, 0.75, 0.82, 0.70, and 0.71, respectively.

**Table 3:** New order proposed for the areas in the user satisfaction survey, according to biserial analysis

Question number	Variables	α Cronbach
	Total survey	0.88
	Area 1. Quality of attention of the unit of Teleneurology	0.80
6	I felt comfortable talking to my specialist doctor through a camera and a microphone	
7	Talking to my specialist doctor. through a camera and a microphone was as effective as in person	
8	During my Telemedicine care it was easy for me to explain my health problem to my specialist doctor	
12	The quality of the image and sound were adequate to talk to my specialist doctor	
14	In general. my attention by Telemedicine was helpful to me	
18	For my future controls I will prefer to continue using Telemedicine	
	Area 2: Patient perception about the service brought by the unit of Teleneurology	0.75
1	In general I am satisfied with the care received in Telemedicine	
2	In general my family is satisfied with the care received in Telemedicine	
3	Telemedicine helps me to know my state of health	
4	Telemedicine helps me know how to improve my health status	
5	Telemedicine allows me to better follow the recommendations and indications of my specialist doctor	
	Area 3: relationship of the patient with the medical team	0.82
13	The general doctor who accompanied me in person helped me during my Telemedicine consultation	
20	My specialist doctor showed concern in solving my health problem during Telemedicine care	
22	The general practitioner who accompanied me in person during the Telemedicine service was able to answer my questions	
23	The general practitioner who accompanied me in person during the Telemedicine care could answer the questions of my specialist doctor	
	Area 4: Relationship of the patient with the specialist doctor	0.70
9	My specialist doctor has identified my health problem through Telemedicine	
10	I have been informed of my right to privacy of my personal and medical information included in Telemedicine	
16	I prefer Telemedicine because it is easier to go to the doctor’s office than to go to the hospital	
17	I prefer Telemedicine because it is cheaper to go to the office than to go to the hospital	
19	My specialist doctor was able to answer my questions through Telemedicine	
	Area 5: Trust in the Teleneurology unit	0.71
11	I trust that my personal information and privacy will be protected after my attention by Telemedicine	
21	I trust the instructions of my specialist doctor during my Telemedicine care	
	Question removed	
15	The time with a specialist is faster by Telemedicine	

### Discussion

Teleneurology arises as an attractive tool to address some of the deficits in the access to timely healthcare for adult neurological patients. In Chile, the Hospital Las Higueras Talcahuano (HHT) has pioneered the implementation of a Teleneurology program with the goal of increasing access of adult patients to remote neurological care in out-patient procedures since 2015. Since there was no survey evaluating patient perception of healthcare quality for the specific area of Teleneurology in the Spanish language, the present 23-item questionnaire was originally designed based on published reports of surveys in the English language and some guidelines for Spanish surveys in the telemedicine field [9–15].

Following refining this survey for face validity by five neurology medical specialists, it was applied to 167 patients enrolled in the HHT Teleneurology program between September 2018 and February 2019. Statistical analyses of data resulting from this study showed high reliability and internal consistency of the designed questionnaire. In addition, the biserial correlation analysis showed that 22 of the 23 items can be more appropriately rearranged to assess five different areas related to quality of care, patient satisfaction, and patient relationship with and trust in the medical personnel involved during the Teleneurology consultation (Additional file 2).

In agreement with the results found on the present study, previously published reports indicate that telemedicine interventions do not hinder the development of the link between the patient and his/her specialist doctor, with high patient satisfaction [4, 16–18]. However, no definitive conclusions should be drawn regarding the quality of care of patients enrolled in the HHT Teleneurology program until a greater scale study can be performed.

In the specific field of Teleneurology, patient satisfaction studies have been conducted Australia, Canada, the United States, France, Israel, Japan, Norway and the United Kingdom [4, 16, 19–21], all non-Spanish speaking countries. Overall, the present pilot study shows that the survey questionnaire in its final form of 22 questions (Table 3) is a reliable tool to evaluate the perception of quality of the HHT Teleneurology program. Validation of the present questionnaire allows for a larger-scale study assessing the impact of this Teleneurology program and its permanent implementation for quality of care vigilance. It also represents the starting point for replicating this experience in other Spanish speaking regions.

## Limitations

1. *Survey bias* We are the only Chilean group in Teleneurology working within the Chilean Public Health system, allowing for no reliable comparison with other programs in the country at the current time.
2. *Size bias* The limited cohort of this pilot study does not allow for comparison of this program user satisfaction with that of the Chilean Public Health system or other telemedicine programs until a larger-scale study is conducted.

## Additional files

**Additional file 1.** Supplementary tables.

**Additional file 2.** Raw data.

## Data Availability

The dataset supporting the conclusions of this article is included within the article and its additional file.

## Abbreviations

HHT: Hospital las Higueras of Talcahuano
SST: Health Service of Talcahuano (SST) of the Ministry of Health
