# Supplementary material for: Validation of a patient satisfaction survey of the Teleneurology program in Chile
Source: BMC Res Notes. 2019 Jun 25;12:359. doi: 10.1186/s13104-019-4358-1 (PMC6593588; doi:10.1186/s13104-019-4358-1)
Supplement: Supplementary file 1 — Additional file 1. Supplementary tables. [file 13104_2019_4358_MOESM1_ESM.docx]

**TABLE S1:** Inclusion and exclusion criteria for potential patients of the Teleneurology Unit of the Hospital Las Higueras de Talcahuano.

| **Inclusion criteria:** According to the reference and counter-referral protocol of the Hospital Las Higueras de Talcahuano for primary health care. The patients are referred with a complete study available in primary health care or performed during previous in-patient admissions. All patient to be included in this study, must have at least one of the 15 below mentioned pathologies. | **Exclusion criteria:** According to the reference and counter-referral protocol of the Hospital Las Higueras de Talcahuano for primary health care. These patients must be immediately derived to the Emergency Room or the Neurology Clinic. According to severity. |
| --- | --- |
| 1- Ischemic or recent hemorrhagic stroke: control and follow-up after hospital discharge.  2.- Chronic daily headache  3.- Non-refractory epilepsy  4.- Dementia syndrome: study phase  5.- Dementias  6.- Polyneuropathies. chronic phase  7.- Chronic movement disorder (eg: essential tremor. chorea. parkinsonian syndrome)  8.- Parkinson's disease  9.- Vertiginous syndrome in chronic phase. as second opinion after evaluation by otorhinolaryngology.  10. Post-traumatic brain injury syndrome (chronic phase).  11.- Peripheral facial paralysis: before diagnostic doubt. in patient without symptoms and / or associated focal focal signs.  12.- Neuropathic pain. chronic phase  13.- Neuropathic pain. acute phase. only if etiology is very evident and treatable in primary care (eg: post herpetic neuralgia)  14.- Myasthenia gravis: chronic control in controlled symptoms with therapy  15.- Acceptance of the patient to use this type of care | 1.- Ischemic or hemorrhagic stroke in the acute phase  2.- Transient ischemic crisis  3.- Thunder or ictal headache  4.- Seizures of recent onset  5.- Convulsive status  6.- Refractory epilepsy  7.- Subacute or rapidly progressive dementia  8.- Delirium  9.- Polyneuropathies. acute polyradiculopathies  10.- Movement disorders of acute onset or extrapyramidal emergencies. myoplegia. hemiplegia. paraplegia or imperfect motility.  11.- Acute vertiginous syndrome with neurological symptoms and warning signs  12.- Cranial brain trauma (acute phase)  13.- Facial paralysis: patient with symptoms and / or associated central focal signs.  14.- Myasthenic syndrome: differential diagnosis study in acute phase  15.- Myasthenia gravis with poor response to usual therapy.  16.- Demyelinating diseases  17.- Mental disability or minors (law 28.584 article 28)  18.- Patient refusal to use this type of care |

**TABLE S2.** Translation of the Study survey to assess the patient satisfaction treated in the Teleneurology Unit of the Hospital Las Higueras de Talcahuano.

|  |  |  |  |  |  |  |  |
| --- | --- | --- | --- | --- | --- | --- | --- |
| **QUESTION #** | **ORIGINAL SPANISH QUESTION** | **TRANSLATED QUESTION** |  |  |  |  |  |
| 1 | En general, estoy satisfecho/a con la atención recibida en Telemedicina. | I am satisfied with the medical care received during my Telemedicine appointment. |  |  |  |  |  |
| 2 | En general, mi familia está satisfecho/a con la atención recibida en Telemedicina. | My family is satisfied with the medical care received during my Telemedicine appointment. |  |  |  |  |  |
| 3 | La Telemedicina me ayuda a conocer mi estado de salud. | Telemedicine helps me to be aware of my health status. |  |  |  |  |  |
| 4 | La Telemedicina me ayuda a saber cómo mejorar mi estado de salud. | Telemedicine helps me to know how to improve my health status. |  |  |  |  |  |
| 5 | La Telemedicina me permite seguir mejor las recomendaciones e indicaciones de mi médico especialista. | Telemedicine allows me to better comply with the specialist doctor recommendations and indications. |  |  |  |  |  |
| 6 | Me sentí cómodo/a al hablar con mi médico especialista a través de una cámara y un micrófono. | I was comfortable talking to the specialist doctor through a camera and a microphone. |  |  |  |  |  |
| 7 | Hablar con mi médico especialista, a través de una cámara y un micrófono, fue tan efectivo como en persona. | Talking to my specialist doctor through a camera and a microphone, was as effective as doing it in person. |  |  |  |  |  |
| 8 | Durante mi atención por Telemedicina me fue fácil explicar mi problema de salud a mi médico especialista. | It was easy for me to explain my health condition to my specialist doctor during my Telemedicine appointment. |  |  |  |  |  |
| 9 | Mi médico especialista ha identificado mi problema de salud a través de Telemedicina. | My specialist doctor has identified my health problem during my Telemedicine appointment. |  |  |  |  |  |
| 10 | He sido informado/a de mi derecho a la privacidad de mi información personal y médica incluida en Telemedicina. | I have been informed of my right to privacy regarding my personal and medical information that was accessed during my Telemedicine appointment. |  |  |  |  |  |
| 11 | Confío que mi información personal y privacidad estarán protegidas después de mi atención por Telemedicina. | I trust that my personal information and privacy will be protected after my Telemedicine appointment. |  |  |  |  |  |
| 12 | La calidad de la imagen y sonido fueron adecuados para hablar con mi médico especialista. | Image and sound qualities were adequate to talk to my specialist doctor. |  |  |  |  |  |
| 13 | El médico general que me acompañó en persona me ayudó durante mi consulta por Telemedicina. | The general doctor who accompanied me in person helped me during my Telemedicine appointment. |  |  |  |  |  |
| 14 | En general, mi atención por Telemedicina me resultó provechosa. | My Telemedicine appointment was helpful. |  |  |  |  |  |
| 15 | La hora con especialista es más rápida por Telemedicina. | The time I have to wait for scheduling the appointment with a specialist is short if it is a Telemedicine appointment. |  |  |  |  |  |
| 16 | Prefiero la Telemedicina porque es más fácil ir al consultorio que ir al hospital. | I would rather schedule a Telemedicine appointment since it is easier to go to the local family clinic than to the hospital. |  |  |  |  |  |
| 17 | Prefiero la Telemedicina porque es más económico ir al consultorio que ir al hospital. | I would rather schedule a Telemedicine appointment since it is cheaper to go to the local family clinic than to the hospital. |  |  |  |  |  |
| 18 | Para mis controles futuros preferiré seguir usando la Telemedicina. | For my future medical care, I would rather schedule Telemedicine appointments. |  |  |  |  |  |
| 19 | Mi médico especialista pudo responder a mis preguntas por Telemedicina | My specialist doctor was able to answer my questions during my Telemedicine appointment. |  |  |  |  |  |
| 20 | Mi médico especialista mostró preocupación en resolver mi problema de salud durante la atención por Telemedicina. | My specialist doctor was engaged in solving my health problem during my Telemedicine appointment. |  |  |  |  |  |
| 21 | Confío en las instrucciones de mi médico especialista durante mi atención por Telemedicina. | I trust the instructions of my specialist doctor during my Telemedicine appointment. |  |  |  |  |  |
| 22 | El médico general que me acompañó en persona durante la atención por Telemedicina pudo responder a mis preguntas. | The general practitioner who accompanied me in person during the Telemedicine appointment was able to answer my questions. |  |  |  |  |  |
| 23 | El médico general que me acompañó en persona durante la atención por Telemedicina pudo responder a las preguntas de mi médico especialista | The general practitioner who accompanied me in person during the Telemedicine appointment could answer the questions made by my specialist doctor |  |  |  |  |  |
|  |  |  |  |  |  |  |  |

**TABLE S3:** Analysis of internal consistency per question of the user satisfaction survey (n = 167)

| **Question Number** | **Variables** | **α when the question is deleted** |
| --- | --- | --- |
|  |  |  |
|  | **Total survey with 23 questions** | 0.88 |
|  |  |  |
| 1 | I am satisfied with the care received in Telemedicine. | 0.87 |
| 2 | My family is satisfied with the care received in Telemedicine. | 0.88 |
|  |  |  |
| 3 | Telemedicine helps me to know my state of health. | 0.88 |
| 4 | Telemedicine helps me know how to improve my health status. | 0.88 |
| 5 | Telemedicine allows me to better follow the recommendations and indications of my specialist doctor. | 0.87 |
|  |  |  |
| 6 | I felt comfortable talking to my specialist doctor through a camera and a microphone. | 0.87 |
| 7 | Talking to my specialist doctor. through a camera and a microphone. was as effective as in person. | 0.87 |
| 8 | During my Telemedicine care it was easy for me to explain my health problem to my specialist doctor. | 0.87 |
| 9 | My specialist doctor has identified my health problem through Telemedicine. | 0.87 |
| 10 | I have been informed of my right to privacy of my personal and medical information included in Telemedicine. | 0.88 |
| 11 | I trust that my personal information and privacy will be protected after my attention by Telemedicine. | 0.88 |
| 12 | The quality of the image and sound were adequate to talk to my specialist doctor. | 0.88 |
| 13 | The general doctor who accompanied me in person helped me during my Telemedicine consultation. | 0.88 |
|  |  |  |
| 14 | My attention by Telemedicine was helpful to me. | 0.87 |
| 15 | The time with a specialist is faster by Telemedicine. | 0.88 |
| 16 | I prefer Telemedicine because it is easier to go to the doctor's office than to go to the hospital. | 0.87 |
| 17 | I prefer Telemedicine because it is cheaper to go to the office than to go to the hospital. | 0.88 |
| 18 | For my future controls I will prefer to continue using Telemedicine. | 0.88 |
| 19 | My specialist doctor was able to answer my questions through Telemedicine | 0.87 |
| 20 | My specialist doctor showed concern in solving my health problem during Telemedicine care. | 0.88 |
| 21 | I trust the instructions of my specialist doctor during my Telemedicine care. | 0.88 |
| 22 | The general practitioner who accompanied me in person during the Telemedicine service was able to answer my questions. | 0.88 |
| 23 | The general practitioner who accompanied me in person during the Telemedicine care could answer the questions of my specialist doctor | 0.88 |

**TABLE S4:** Difficulty index of the patient satisfaction survey questions (n = 167)

| **Question number** | **Variables** | **Difficulty index** | |
| --- | --- | --- | --- |
|  |  |  |  |
|  |  |  | |
| 1 | I am satisfied with the care received in Telemedicine. | 0.87 |  |
| 2 | My family is satisfied with the care received in Telemedicine. | 0.67 |  |
| 3 | Telemedicine helps me to know my state of health. | 0.81 |  |
| 4 | Telemedicine helps me know how to improve my health status. | 0.74 |  |
| 5 | Telemedicine allows me to better follow the recommendations and indications of my specialist doctor. | 0.83 |  |
| 6 | I felt comfortable talking to my specialist doctor through a camera and a microphone. | 0.79 |  |
| 7 | Talking to my specialist doctor. through a camera and a microphone. was as effective as in person. | 0.76 |  |
| 8 | During my Telemedicine care it was easy for me to explain my health problem to my specialist doctor. | 0.81 |  |
| 9 | My specialist doctor has identified my health problem through Telemedicine. | 0.81 |  |
| 10 | I have been informed of my right to privacy of my personal and medical information included in Telemedicine. | 0.85 |  |
| 11 | I trust that my personal information and privacy will be protected after my attention by Telemedicine. | 0.86 |  |
| 12 | The quality of the image and sound were adequate to talk to my specialist doctor. | 0.83 |  |
| 13 | The general doctor who accompanied me in person helped me during my Telemedicine consultation. | 0.92 |  |
| 14 | My attention by Telemedicine was helpful to me. | 0.92 |  |
| 15 | The time with a specialist is faster by Telemedicine. | 0.87 |  |
| 16 | I prefer Telemedicine because it is easier to go to the doctor's office than to go to the hospital. | 0.86 |  |
| 17 | I prefer Telemedicine because it is cheaper to go to the office than to go to the hospital. | 0.77 |  |
| 18 | For my future controls I will prefer to continue using Telemedicine. | 0.78 |  |
| 19 | My specialist doctor was able to answer my questions through Telemedicine | 0.90 |  |
| 20 | My specialist doctor showed concern in solving my health problem during Telemedicine care. | 0.92 |  |
| 21 | I trust the instructions of my specialist doctor during my Telemedicine care. | 0.92 |  |
| 22 | The general practitioner who accompanied me in person during the Telemedicine service was able to answer my questions. | 0.91 |  |
| 23 | The general practitioner who accompanied me in person during the Telemedicine care could answer the questions of my specialist doctor | 0.93 |  |

**TABLE S5:** Biserial correlation of the questions included in the user satisfaction survey (n = 167)

| **Question number** | **Variables** | **Biserial correlation** | |
| --- | --- | --- | --- |
|  |  |  |  |
| 1 | I am satisfied with the care received in Telemedicine. | 0.62 | |
| 2 | My family is satisfied with the care received in Telemedicine. | 0.52 | |
| 3 | Telemedicine helps me to know my state of health. | 0.51 | |
| 4 | Telemedicine helps me know how to improve my health status. | 0.54 | |
| 5 | Telemedicine allows me to better follow the recommendations and indications of my specialist doctor. | 0.57 | |
| 6 | I felt comfortable talking to my specialist doctor through a camera and a microphone. | 0.55 | |
| 7 | Talking to my specialist doctor. through a camera and a microphone. was as effective as in person. | 0.66 | |
| 8 | During my Telemedicine care it was easy for me to explain my health problem to my specialist doctor. | 0.63 | |
| 9 | My specialist doctor has identified my health problem through Telemedicine. | 0.56 | |
| 10 | I have been informed of my right to privacy of my personal and medical information included in Telemedicine. | 0.47 | |
| 11 | I trust that my personal information and privacy will be protected after my attention by Telemedicine. | 0.48 | |
| 12 | The quality of the image and sound were adequate to talk to my specialist doctor. | 0.45 | |
| 13 | The general doctor who accompanied me in person helped me during my Telemedicine consultation. | 0.58 | |
| 14 | My attention by Telemedicine was helpful to me. | 0.66 | |
| 15 | The time with a specialist is faster by Telemedicine. | 0.47 | |
| 16 | I prefer Telemedicine because it is easier to go to the doctor's office than to go to the hospital. | 0.60 | |
| 17 | I prefer Telemedicine because it is cheaper to go to the office than to go to the hospital. | 0.52 | |
| 18 | For my future controls I will prefer to continue using Telemedicine. | 0.59 | |
| 19 | My specialist doctor was able to answer my questions through Telemedicine | 0.57 | |
| 20 | My specialist doctor showed concern in solving my health problem during Telemedicine care. | 0.60 | |
| 21 | I trust the instructions of my specialist doctor during my Telemedicine care. | 0.45 | |
| 22 | The general practitioner who accompanied me in person during the Telemedicine service was able to answer my questions. | 0.42 | |
| 23 | The general practitioner who accompanied me in person during the Telemedicine care could answer the questions of my specialist doctor | 0.38 | |
|  | |  |  |
